# Immunomodulation of J774A.1 Murine Macrophages by *Lactiplantibacillus plantarum* Strains Isolated From the Human Gastrointestinal Tract and Fermented Foods

**DOI:** 10.3389/fmicb.2020.557143

**Published:** 2021-01-12

**Authors:** Natalia Garcia-Gonzalez, María A. Nuñez-Sanchez, Miguel Villoria Recio, Natalia Battista, Cormac G. M. Gahan, Aldo Corsetti

**Affiliations:** ^1^Faculty of Bioscience and Technology for Food, Agriculture and Environment, University of Teramo, Teramo, Italy; ^2^School of Microbiology, University College Cork, Cork, Ireland; ^3^APC Microbiome Ireland, University College Cork, Cork, Ireland

**Keywords:** cytokines, food-borne microbes, health-promoting bacteria, immune system, *Lactiplantibacillus plantarum*, macrophages

## Abstract

*Lactobacillus plantarum* species (recently re-named *Lactiplantibacillus (Lpb.) plantarum* subsp. *plantarum*) can be isolated from both either the mammalian gut or specific fermented foods where they may be present at high concentrations. Whilst *Lpb. plantarum* strains have been proposed as potential probiotic candidates, the ability of resident strains consumed in fermented foods to interact with the host is unclear. The main objective of this study was to investigate the cellular location and ability of three different food-borne *Lpb. plantarum* strains isolated from different sources (table olives and cheese) to modulate the immune response of a murine macrophage-like cell line (J774A.1). For that purpose, macrophages were exposed to the three different *Lpb. plantarum* strains for 24 h and the expression of a panel of genes involved in the immune response, including genes encoding pattern-recognition receptors (TLRs and NLRs) and cytokines was evaluated by qRT-PCR. We also utilized chemical inhibitors of intracellular pathways to gain some insight into potential signaling mechanisms. Results showed that the native food strains of *Lpb. plantarum* were able to modulate the response of J774A.1 murine macrophages through a predominately NOD signaling pathway that reflects the transient intracellular location of these strains within the macrophage. The data indicate the capacity of food-dwelling *Lpb. plantarum* strains to influence macrophage-mediated host responses if consumed in sufficient quantities.

## Introduction

The original genus *Lactobacillus* (*L.*) comprised a heterogeneous group of non-sporeforming, non-motile and rod-shaped bacteria (Plot et al., 2014). However, a recent phylogenetic and ecological revaluation of the genus has led to reclassification of *Lactobacillus* species into 25 proposed genera (Zheng et al., 2020). In particular *Lactobacillus plantarum* has been reclassified as *Lactiplantibacillus plantarum* subsp. *plantarum (Lpb. plantarum). Lpb. plantarum* is a microaerophilic gram-positive bacterium, that is encountered in a wide variety of ecological niches including the gastrointestinal tract and fermented foods (Corsetti et al., 2018). Moreover, *Lpb. plantarum* has been used as a starter culture in food fermentation processes due to its organoleptic fermentative properties and a capacity to produce lactic acid and other antimicrobial compounds (Seddik et al., 2017; Behera et al., 2018). We have recently shown that *Lpb. plantarum* strains are likely to be consumed at high concentrations in table olives where they are one of the predominant species (10^7^–10^8^ CFU/g) (Hurtado et al., 2012; Heperkan, 2013; Perpetuini et al., 2020). However, the potential impact of these autochthonous food strains on the host, when either consumed directly or re-employed as starter cultures, is currently unclear.

In the last century, *Lpb. plantarum* strains have been widely investigated not only for their functional properties but also as potential probiotics. Probiotics are defined as live microorganisms that, when administered in adequate amounts, confer a health benefit on host health (Hill et al., 2014). Bacteria commonly associated with probiotic effects usually have the ability to adhere to intestinal cells, produce beneficial metabolites such as short-chain fatty acids, modulate the immune system and compete with pathogens for adhesion sites (de Vrese and Schrezenmeir, 2008; Wells, 2011). Immune modulatory properties of some *Lactobacillus* strains have been described in both animal studies and clinical trials. However, the exact mechanisms underlying such effects and the inter-strain variation in these properties are not fully understood (Corthésy et al., 2007). According to the latest evidence, the definition of probiotics regarding their immunomodulatory properties could be extended to *“live microorganisms, that when included in foods can influence the composition and activity of the gut microbiota, modulate the inflammatory response, improve the non-specific intestinal barrier, and reinforce or modulate the mucosal and the systemic immune responses”* (Dong et al., 2010; Markowiak and Śliżewska, 2017). Beneficial effects of probiotic strains in modulating the immune system could impact positively in inflammatory processes, which have been described to be involved in a number of chronic diseases and disorders including osteoporosis, cardiovascular disease, insulin resistance and diabetes (Cani et al., 2008; Kang and Im, 2015; Ryan et al., 2019). Several studies have demonstrated that probiotics are able to stimulate the release of pro-inflammatory cytokines by activation of pattern recognition receptors (PRRs) (Melmed et al., 2003; Lee et al., 2006; Ghadimi et al., 2010; Macho Fernandez et al., 2011; Zhong et al., 2012). The use of *in vitro* models has been a valuable tool to elucidate the effect on the immune system by *Lpb. plantarum* strains. They have been described to reduce the expression of genes involved in the pro-inflammatory response by activating the expression of TLR2 (Pathmakanthan et al., 2004; Paolillo et al., 2009; Bäuerl et al., 2013). For instance, *L. plantarum* Lp62 has been described to inhibit the inflammatory stimulation in epithelial cells and macrophages by modulating the release of TNF-α, IL-1ß, and IL-17 (Ferreira dos Santos et al., 2016). However, the cross-talk between host cells and bacteria belonging to the *Lactobacillus* genus seems to be strain-dependent and the observed effects for a specific strain cannot be extrapolated to other bacteria within the same species, and thus, it is crucial to evaluate the potential probiotic effects of individual strains (Ivec et al., 2007; Tallon et al., 2007).

The aim of this study was to evaluate the effect of three *Lpb. plantarum* isolated from different sources in the early cytokine response by modulating PRRs in a murine macrophage cell line. Properties including cytotoxicity, adhesion, intracellular localization, and the cross-talk between bacteria and host cells were evaluated. Moreover, specific inhibitors of the upstream kinases, interleukin-1 receptor-associated kinase 4 (IRAK4) and receptor-interacting-serine/threonine-protein kinase 2 (RIP2) and specific inhibitors of the phagocytosis, cytochalasin D (Cyt.D) and dehydroxymethylepoxyquinomicin (DHMEQ), were used in order to elucidate the exact PRRs signaling pathways activated by *Lpb. plantarum* strains. This study demonstrates that *Lpb. plantarum* strains that are found in natural food environments are capable of stimulating macrophage cytokine responses through a NOD-dependent signaling mechanism reflective of their transient intracellular location. Whilst further *in vivo* studies are warranted, the current study suggests that naturally occurring food-resident *Lpb. plantarum* strains may have the potential to elicit host immune effects when consumed at adequate levels.

## Materials and Methods

### Bacterial Strains

Food-borne *Lpb. plantarum* strains C904 and LT52 isolated from different fermented foods (table olives and raw-milk cheese, respectively) belonging to our collection (University of Teramo), the probiotic strain IMC513, isolated from the gastrointestinal tract of human (Synbiotec, Camerino, Italy) and *Listeria (L.) innocua* from our collection (University College Cork) were used in this study. Whilst all *Lpb. plantarum* strains were grown in microaerophilic conditions in MRS broth (Oxoid, Basingstoke, United Kingdom), *L. innocua* was grown on Brain Heart Infusion (BHI) broth (Oxoid, Basingstoke, United Kingdom) in aerobic conditions. All species were grown at 37°C and subcultured once before each experiment.

### Cell Line and Culture Conditions

The J774A.1 murine macrophage cell line was purchased from the American Tissue Culture Collection (ATCC, Rockville, United States). Cells were cultured as recommended by the ATCC in Dulbecco’s Modified Eagle’s medium (DMEM; 4.5 g/L D-glucose, Sigma-Aldrich Ltd., Wicklow, Ireland) supplemented with 10% v/v heat-inactivated foetal bovine serum (FBS) (Sigma-Aldrich Ltd., Wicklow, Ireland). Cells were maintained at 37°C in an incubator under a 5% CO_2_ and 95% relative humidity. Prior to the experiments, cells were detached from the flask using a scraper, centrifuged at 1,500 rpm for 4 min at room temperature (RT) and resuspended in antibiotic-free growth media. Cells were counted using a hemocytometer and seeded at a density of 50,000 cells/cm^2^ for 24 h prior to incubation with *Lpb. plantarum* strains. Control cells were also run in parallel and subjected to the same changes of medium.

### Viability Assays

After 24 h of exposure of the macrophages to each strain of *Lpb. plantarum*, cell viability was evaluated using the CellTiter-Glo^®^ luminescent cell viability assay (Promega, MyBio Ltd., Ireland) following the manufacturer’s instructions. Briefly, cells were exposed to each *Lpb. plantarum* strains (ratio bacteria/macrophage 10:1 and 100:1) for 24 h. After the treatment, cell media was removed, and wells were washed three times to remove the bacteria in the supernatant prior to the addition of fresh medium. Plates were then placed at RT for 30 min to equilibrate the plate and then, an equal volume of CellTiter-Glo^®^ was added to each well. The contents were mixed for 5 min using an orbital shaker at 200 rpm. The plates were allowed to stabilize for 15 min at RT and luminescence was measured using a BioTek Synergy two microplate reader (BioTek Instruments, Winooski, VT, United States). All experiments were performed in triplicate.

In order to confirm these data, cell viability was further determined by flow cytometry using propidium iodide (PI, R&D Biosciences, Minneapolis, MN, United States) staining. Cells were cultured in 12-well plates and treated as described above. To detach the cells, the macrophages were placed on ice for 15 min under constant shaking at 200 rpm. Then, media was collected in a 15 mL falcon tube and 0.5 mL of TrypLE Express (Gibco, Biosciences, Dublin, Ireland) were added to each well. Plates were incubated for 10 min on ice under constant shaking (200 rpm) and then collected in the same tube and centrifuged at 1,500 rpm for 5 min. Cells were washed twice with Flow Cytometry Staining Buffer (FCSB) containing PBS with 2% FBS and centrifuged at 1,500 rpm for 5 min. Cells were then resuspended in 100 μL of FCSB containing 5 μL of PI and incubated 15 min at RT protected from light. Cell viability was analyzed by flow cytometry using a BD Accuri C6 cytometer (Becton Dickinson, NJ, United States).

### Adhesion and Phagocytosis Assays

The ability of bacteria to adhere to murine macrophages was investigated as previously described by Vargas-García and colleagues (Vargas García et al., 2015) with some modifications. Briefly, cells were incubated in 96-well plates and *Lpb. plantarum* strains were added at a density of 10^6^ CFU/ml (ratio bacteria/macrophages 10:1). The bacteria were allowed to adhere to the cells for 4 h at 37°C. After that, cells were detached and plated in MRS agar. The percentage of adherent bacteria was calculated by comparing the total number of bacterial colonies counted to the number of bacteria added. In order to calculate the total cell-associated bacteria, macrophages were lysed with 0.2% Triton X-100 for 20 min prior to preparation of the dilution series. The percentage of total associated bacteria was calculated by comparing the total number of bacterial colonies counted after the macrophages were lysed to the number of bacteria added. Moreover, in order to calculate the number of internalized bacteria, phagocytosis assay was performed by stopping the internalization with ice-cold PBS and killing the remaining extracellular bacteria by addition of gentamicin (100 μg/mL) for 2 h. After the incubation, cells were lysed with 0.2% Triton X-100 and plated in MRS agar plates. All the experiments were performed in triplicate.

### Microscopic Visualization of *Lpb. plantarum* Strains

J774A.1 macrophages were seeded on top of a microscope slide inside 60 mm petri dishes at a density of 0.8 × 10^6^ cells overnight. Upon adherence, cells were exposed to *Lpb. plantarum* strain C904, LT52, or IMC513 at a ratio of 10:1 (bacteria/macrophages) for 6 h. In brief, single colonies of each strain were picked, diluted in PBS and used to cover J774A.1 cells on individual microscope slides. Both cell and bacterial staining was performed using Shandon^TM^ Kwik-Diff^TM^ Giemsa stains (ThermoFisher, Waltham, MA, United States). Briefly, the microscope slides were dipped orderly 15 times in methanol, eosin and finally methylene Blue, washed with tap water. Samples were let to dry, mounted with mounting media (Thermo Fisher, Waltham, MA, United States) and hold using a coverslide. A light field microscope (Zeiss, Germany) was used to take pictures at 100× magnification.

### Quantitative Real-Time Reverse Transcriptase-Polymerase Chain Reaction (qRT-PCR) Analysis

Expression of inflammatory markers in macrophages was evaluated through the analysis of gene expression of a panel of cytokines and receptors involved in the microbial interaction and inflammatory response (Table 1). Briefly, J774A.1 macrophages were plated in triplicated in 12-well plates and cultured with the *Lpb. plantarum* strains (10^6^ CFU/ml) (ratio bacteria/macrophages 10:1). After the incubation, cells were lysed in the plates and total mRNA was isolated using an Isolate II RNA mini kit (Bioline, Medical Supply Company Ltd., Dublin, Ireland). mRNA was eluted in RNAse-free water and checked for concentration and purity using a Nanodrop spectrophotometer system (ND-1000 3.3 Nanodrop Technologies, United States). cDNA was obtained from mRNA following reverse transcription according to the manufacturer’s instructions (NZY First-Strand cDNA Synthesis Kit, Nzytech, Lisbon, Portugal). The results were normalized against β-actin gene expression.

**TABLE 1:** List of primers used in the study.

Gene Symbol	Official full name	Gene ID	mRNA Accession	Forward	Reverse	Probe
*Actb*	Actin, beta	11461	NM_007393	aaggccaaccgtgaaaagat	gtggtacgaccagaggcatac	#56
*Il6*	Interleukin 6	16193	NM_031168	gctaccaaactggatataatcagga	ccaggtagctatggtactccagaa	#6
*Il10*	Interleukin 10	16153	NM_010548	cagagccacatgctcctaga	tgtccagctggtcctttgtt	#41
*Ifn*	Interferon gamma	15978	NM_008337	atctggaggaactggcaaaa	ttcaagacttcaaagagtctgagg	#21
*Tnf*	Tumor necrosis factor	21926	NM_013693	ctgtagcccacgtcgtagc	ttgagatccatgccgttg	#25
*Nod1*	Nucleotide-binding oligomerization domain containing 1	107607	NM_172729	ccttgctgagagtcaccgta	ctgcctttcattgctgacc	#81
*Nod2*	Nucleotide-binding oligomerization domain containing 2	257632	NM_145857	ccctagcactgatgctgga	ccccttcgtcacagatatgg	#13
*Tlr2*	Toll like receptor 2	24088	NM_011905	ggggcttcacttctctgctt	agcatcctctgagatttgacg	#50
*Tlr4*	Toll like receptor 4	21898	NM_021297	ggactctgatcatggcactg	ctgatccatgcattggtaggt	#2
*Tlr5*	Toll like receptor 5	53791	NM_016928	ctggagccgagtgaggtc	cggcaagcattgttctcc	#1
*Tlr9*	Toll like receptor 9	81897	NM_031178	gagaatcctccatctcccaac	ccagagtctcagccagcac	#79

### Macrophage Stimulation Assay With RIP2 and IRAK4 Inhibitors

J774A.1 macrophages were seeded in 96-well plates and incubated overnight at 37°C and 5% CO_2_ to allow adherence. Cells were treated with PF 06650833 (100 nM) (TOCRIS, Abingdon, United Kingdom), a specific inhibitor of IRAK4, or Gefitinib (10 μM) (InvivoGen, San Diego, CA, United States), an inhibitor of RIP2 for 1 h before being stimulated by bacteria at a concentration of 10^6^ CFU/ml (ratio bacteria/macrophages 10:1) for 24 h (Seganish, 2016). The release of TNF-α and IL-6 was evaluated using enzyme-linked immunosorbent assays (ELISA).

### Enzyme-Linked Immunosorbent Assay

Levels of TNF-α and IL-6 were measured using Murine TNF-α and IL-6 Mini ABTS ELISA Development Kit (Peprotech, Inc., London, United Kingdom) and analyzed using a microplate reader. When appropriate, evaluation of potential signaling pathways was performed on murine macrophages pre-treated with inhibitor of IRAK4 and Gefitinib for 1 h to inhibit IRAK4 and RIP2, and 2 μM of Cyt.D or 10 μg of DHMEQ for 24 h to inhibit phagocytosis (Suzuki and Umezawa, 2006; Weibel et al., 2012) prior to 24 h of bacterial stimulation. Following this incubation, supernatants were collected and analyzed following the manufacturer’s instructions. J774A.1 murine macrophages without bacterial treatment and without inhibitors pre-treatment were used as a control.

### Statistical Analysis

Data were analyzed using the Prism 5.0 program (GraphPad Software Inc., La Jolla, CA, United States) using the one-way analysis of variance (ANOVA) followed by Bonferroni’s *post hoc* analysis. ELISA data were assessed by Student’s *t*-test. All values are given as mean ± SE. A level of *p* < 0.05 was considered statistically significant.

## Results

### Microbial Impact of *Lpb. plantarum* Strains on J774A.1 Murine Macrophages

**FIGURE 1:**
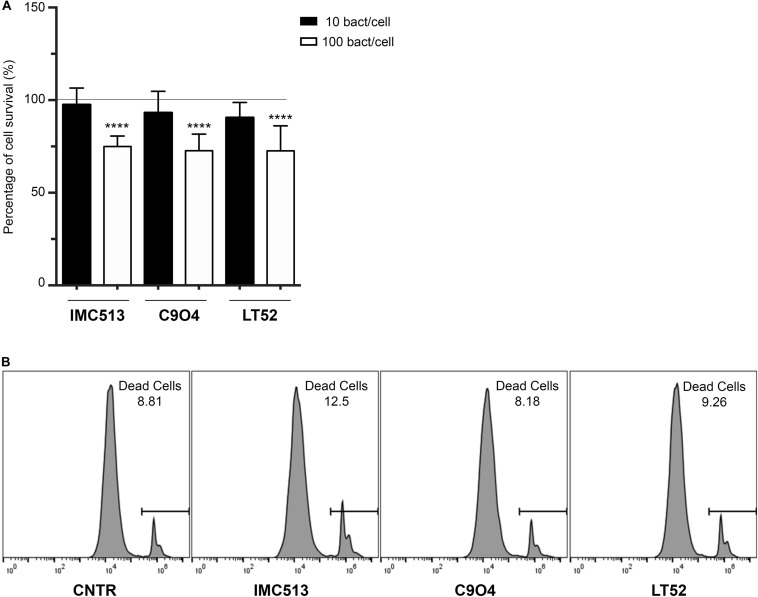
Evaluation of cellular metabolic activity in murine macrophages J774A.1 cells incubated with three different *Lpb. plantarum* strains. **(A)** Macrophage viability was evaluated by measuring the ATP content with Promega Cell-Titer-Glo^®^. Data are shown as mean ratio (percentage ± SD) of absorbance in the samples well (OD of macrophages with bacteria) and negative control (OD of macrophages). **(B)** Number of viable cells evaluated by flow cytometry using the PI staining. Representative values of death cells are given as percentage. Statistical analysis was conducted using One-way ANOVA followed by Bonferroni’s *post hoc* analysis. Values are expressed as mean ± SD, ****p < 0.0001.

The potential impact of three *Lpb. plantarum* strains on viability of J774A.1 murine macrophages was assessed by the luminescent CellTiter-Glo assay. In order to evaluate the potential toxicity of bacteria on J774A.1, cell viability was tested using two different concentrations of bacteria 10^6^ CFU/mL and 10^7^ CFU/mL. After 24 h incubation, our results showed that the lowest concentration tested had no significant impact on cell viability with all values above 90%. On the other hand, cells exposed to *Lpb. plantarum* strains at a concentration of 10^7^ CFU/mL showed a significant decrease (*p* < 0.001) in cell viability after 24 h exposure. The percentage of survival of macrophages after the treatment is shown in Figure 1A.

In order to confirm this data and that the observed values correspond only to the macrophage’s metabolic activity and not to the combined signal of macrophages and remaining bacteria, cell viability was also evaluated by flow cytometry using PI staining (Figure 1B). Our results showed that the percentage of death cells in the untreated cells reached a mean value of 8.6%. After the exposure to the different *Lpb. plantarum* strains, the results demonstrated that the strains had a similar percentage of cell death with C904 strain showing the lowest percentage with 7.9% of cell death while the probiotic strain IMC513 induced the highest cell death with a mean value of 11.7% although it was not significant.

### Adhesion Efficiency of *Lpb. plantarum* Strains to Murine Macrophages

**FIGURE 2:**
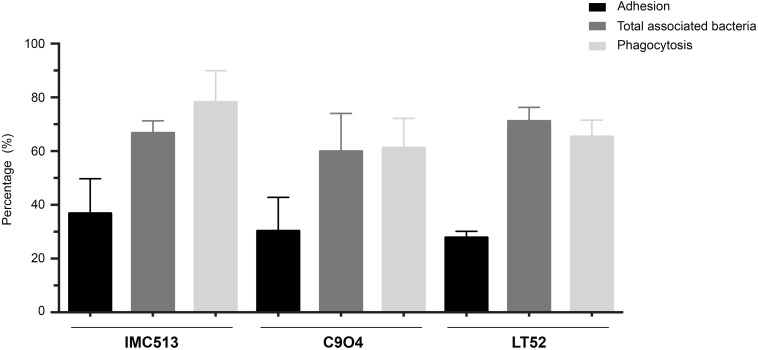
Adhesion and phagocytosis of three *Lpb. plantarum* strains by J774A.1 murine macrophages. For adhesion assays, *Lpb. plantarum* strains were co-incubated with macrophages and the percentage of adherent bacteria was determined by plating on MRS. The total amount of macrophage-associated bacteria was performed by lysing of the macrophages after the incubation. Finally, for phagocytosis assays remaining extracellular bacteria were killed to only evaluate internalized bacteria.

The ability of *Lpb. plantarum* strains to adhere to J774.A1 murine macrophages was tested at the selected concentration of 10^6^ CFU/mL through bacterial enumeration by plating on MRS agar. The adhesion of the probiotic strain IMC513 showed the highest values with a mean of 36.83 ± 6.46%. On the other hand, the LT52 strain showed the lowest values, although differences were not statistically significant between any of the three *Lpb. plantarum* strains (Figure 2). The total amount of macrophage-associated bacteria was evaluated by lysing of the macrophages before enumeration in order to liberate internalized bacteria. Results showed that most of the bacteria were internalized after 4 h incubation with macrophages (Figure 2). These results were further confirmed through the evaluation of the bacteria phagocytised following an antibiotic treatment in order to kill all the remaining bacteria in the media and those that were adherent but not internalized. Our results were in line with the observed results of adherence and total bacteria. To further verify the internalization of the bacteria, we performed a microscopic visualization using Shandon^TM^ Kwik-Diff^TM^ Giemsa stains (Supplementary Figure 1). Microscopic evaluation showed the internalization of the bacteria by macrophages and their apparent localization in the cytoplasm of the macrophages. We appreciate that it is unclear whether bacteria remain internalized or are freely present in the cytoplasm however bacteria are clearly cell-associated and gentamicin protection assays indicate that they remain viable (Figure 2). Regardless the origin of the strains, the ability to internalized bacteria by macrophages did not show significant differences, with internalization rates ranging from 61.22% (C904) to 78.24% (IMC513) (Figure 2).

### *Lpb. plantarum* Modulation of Genes Involved in Inflammation on Murine Macrophages

**FIGURE 3:**
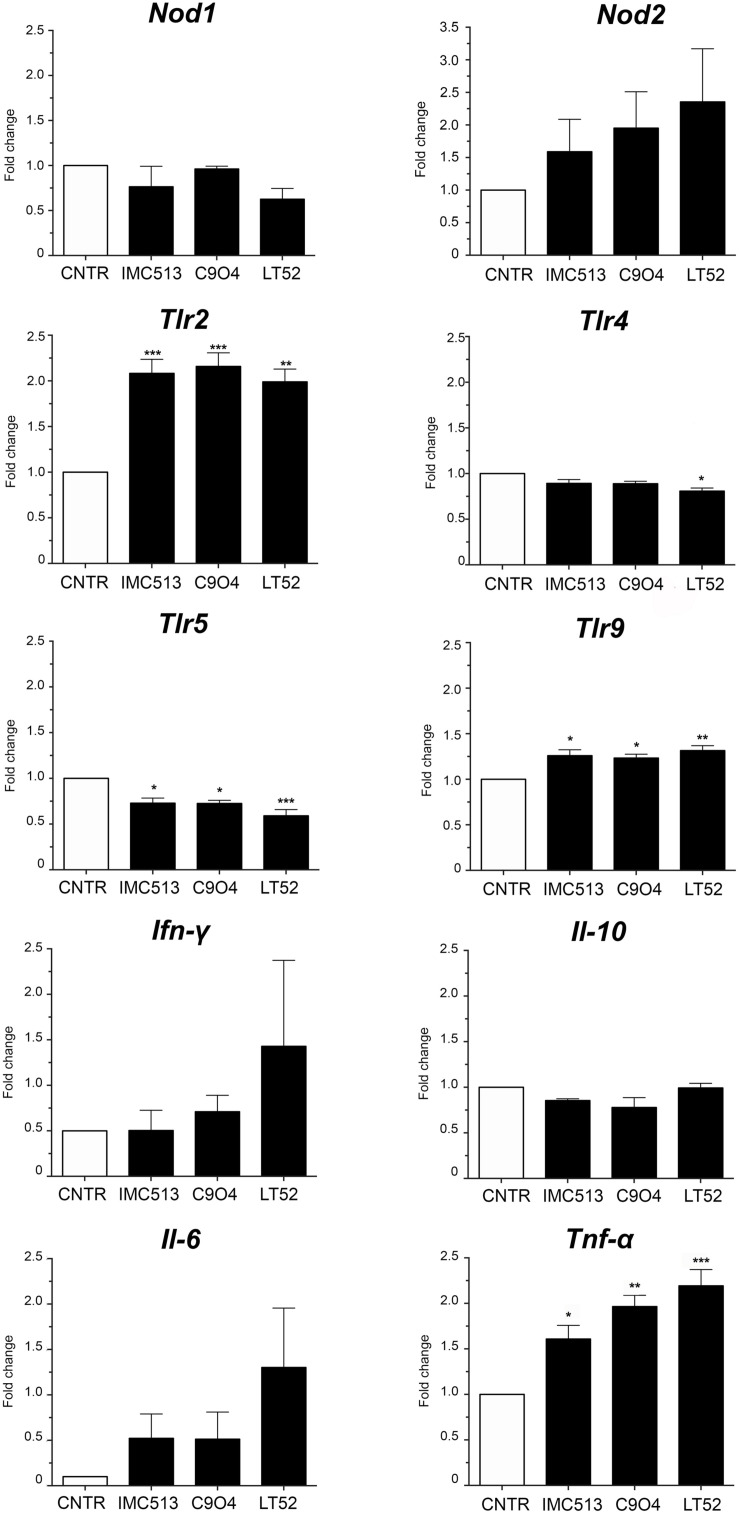
The expression level of TLRs, NLRs and cytokines. After 24 h incubation, the gene expression of NLRs (*Nod1* and *Nod2*), TLRs (*Tlr2*, *Tlr4*, *Tlr5*, and *Tlr9*) and cytokines (*Ifn-γ*, *Il-10*, *Il*-6 and *Tnf-α*) was evaluated by qRT-PCR in macrophages incubated with *Lpb. plantarum* strains. Statistical analysis was conducted using One-way ANOVA followed by Bonferroni’s *post hoc* analysis. Values are expressed as mean ± SD, *p < 0.05, **p < 0.01, ***p < 0.001.

Based on the high adhesion efficiency of the three strains we then evaluated the potential immunomodulatory impact of the three *Lpb. plantarum* strains IMC513, C904, and LT52 on J774A.1 murine macrophages. For that purpose, we selected a panel of genes encoding proteins involved in the recognition of microbe associated molecular patterns and in the inflammatory response. To evaluate the recognition of the strains we analyzed expression of two of the major types of PRRs; leucine-rich repeat containing receptors (NLRs) *Nod1* and *Nod2*, and the toll-like receptors (TLRs) *Tlr2*, *Tlr4*, *Tlr5*, and *Tlr9*. The results of qRT-PCR (Figure 3) indicated that there are differences in the expression of both TLRs and NLRs receptors after incubation with different *Lpb. plantarum* strains. The expression levels of *Nod2, Tlr2* and *Tlr9* were increased to statistically significant levels in the case of TLRs when exposed to all *Lpb. plantarum* strains (*p* < 0.05). On the other hand, the expression levels of *Nod1*, *Tlr4* and *Tlr5* seemed to be repressed although of these, but only *Tlr5* appeared downregulated significantly (Figure 3). We appreciate that some of these alterations in gene expression are subtle (less than 0.5 to 1-fold) and the biological significance of the findings will be the subject of further work.

Regarding inflammatory biomarkers, the gene expression of *Ifn-γ*, *Il-10*, *Il-6*, and *Tnf-α* was evaluated after incubation with *Lpb. plantarum* strains (Figure 3). Results showed an increase of mRNA expression levels of *Ifn-γ* and *Il-6*, while the levels of *Il-10* remained non-significant when compared to the control. On the other hand, the expression of *Tnf-α* was significantly increased with all the strains with LT52 promoting the strongest increase.

**FIGURE 4:**
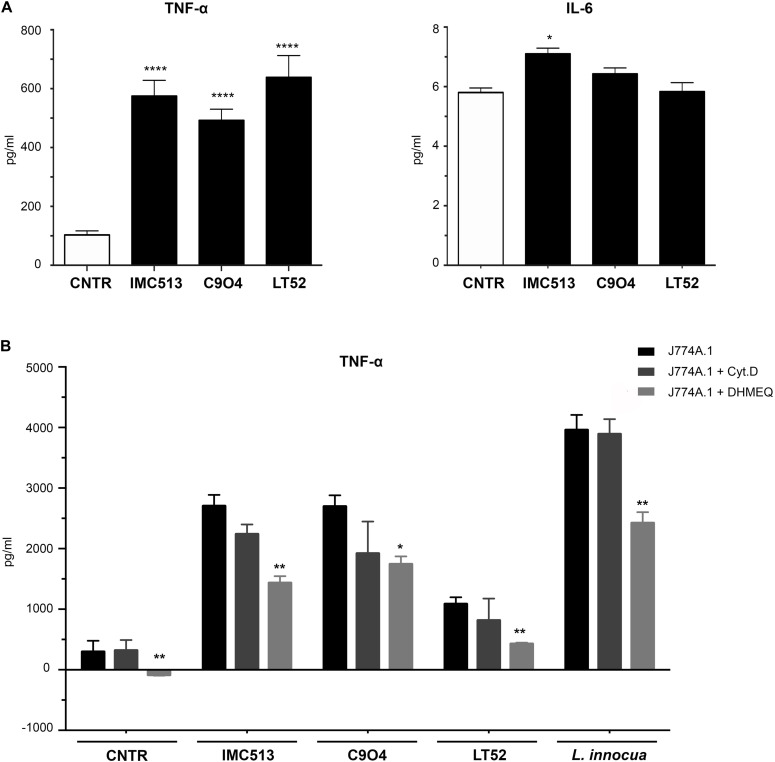
Quantification of the inflammatory response of murine macrophages J774A.1 using ELISA. **(A)** Release of IL-6 was found low, while strong amounts of TNF-α are shown in **(B)**, where **c**ells were seeded and exposed to the phagocytosis inhibitor drugs Cyt.D and DHMEQ for 24 h. Cells were subsequently stimulated with either *Lpb. plantarum* LT52, IMC513, and C904 or *L. innocua* for 24 h and supernatants collected. Values are expressed as mean ± SD and assessed by Student *t*-test, where *p < 0.05 and **p < 0.001.

In order to explore the inflammatory response, the PRR-dependent release of IL-6 and TNF-α was quantified upon macrophage exposure to the *Lpb. plantarum* strains for 24 h using the non-pathogenic, gram-positive *L. innocua* as control. While no substantial response was found in the case of IL-6 (Figure 4A), TNF-α was found to be strongly induced upon exposure to all *Lpb. plantarum* strains in comparison to a control, with IMC513 and C904 strains showing a similar and higher release than LT52 but at lower levels than occurred upon exposure to *L. innocua* (Figure 4B).

To further confirm that the inflammatory response was specifically triggered upon phagocytosis, we confirmed under a microscope that all *Lpb. plantarum* strains could be found internalized in the macrophages at 6 h post-exposure. Indeed, further support was observed by employing the phagocytosis inhibitor Cyt.D or DHMEQ, which also inhibits the LPS-induced Nuclear Factor kappa-light-chain-enhancer of activated B cells (NF-kB) activation (Suzuki and Umezawa, 2006). Here, TNF-α release levels appeared lower upon exposure to Cyt.D but were statistically significant in all strains tested under the effect of DHMEQ only (Figure 4B). Taken together, these results support that the inflammatory response of macrophages to *Lpb. plantarum* exposure is strain specific and that phagocytosis is important to drive a full inflammatory response. The work is broadly supportive of a recent finding that certain strains with probiotic potential may stimulate intracellular NOD pathways (Brown et al., 2017).

### NOD2 but Not TLRs Mediates NF-kb Activation by *Lpb. plantarum* Strains

**FIGURE 5:**
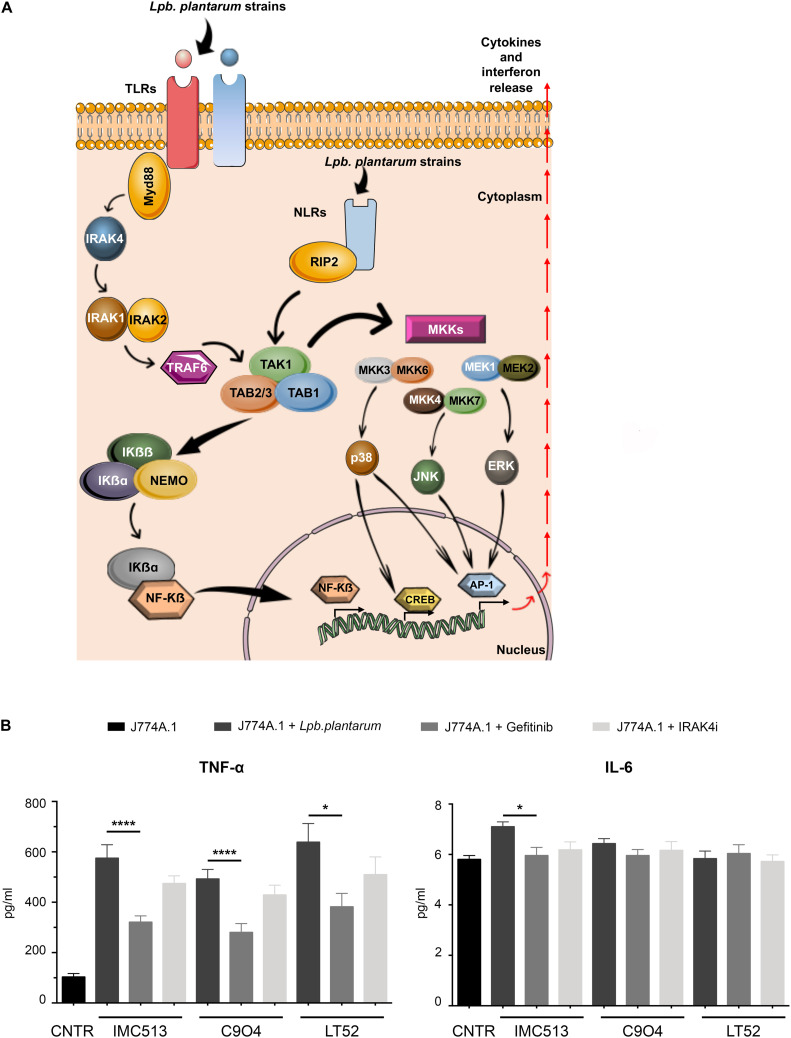
**(A)** Schematic representation of the signaling pathways activated downstream by PRR. This scheme is an adaptation of Plaza-Diaz et al. (2019) contains free available designs from Servier Medical Art templates (https://smart.servier.com/). **(B)** IL-6 and TNF-α content in J774A.1 macrophages treated with specific RIP2 inhibitor and IRAK4 inhibitors following *Lpb. plantarum* stimulation. Briefly, J774A.1 macrophages were seeded and pre-incubated with each inhibitor 1 h before bacterial stimulation. After the co-incubation, supernatants were collected and quantification of TNF-α and IL-6 release was evaluated by ELISA. Values are expressed as mean ± SD. Values were assessed by Student *t-*test comparing data for tests including inhibitors with the controls (stimulated by bacteria but lacking inhibitor). *p < 0.05, ****p < 0.0001.

In order to evaluate the mechanisms involved in the observed immune response and shed light on the nature of the PRR type contributing to macrophage activation upon *Lpb. plantarum* exposure, we performed a test using specific chemical inhibitors. IRAK4 and RIP2 proteins were selected as they are immediate in the downstream pathways activated by PRRs. IRAK4 is involved in Myd88 signaling cascades, the first domain recruited after activation of the TLRs, and plays an essential role in inflammation related disorders (Seganish, 2016), while RIP2 is critical for signal propagation of NLRs (Figure 5A). However, since *Lpb. plantarum* is able to interact with both type of receptors, the implication of each one in the cytokine release remains unclear. Thus, inhibitors of the proteins IRAK4 (IRAK4 inhibitor) and RIP2 (Gefinitib) were used in the study. The inhibition of RIP2 protein by Gefitinib significantly reduced the production of TNF-α induced by each *Lpb. plantarum* strains tested (Figure 5B). This suggests an inter-dependence on the RIP2 adaptor protein for an effective TNF-α response to the bacteria tested. RIP2 is the adaptor protein for NOD1 and NOD2, the decrease in cytokine production could be due to a dependence on signaling through the NOD2 receptor, the expression of which was activated by the *Lpb. plantarum* strains tested (Figure 3). On the other hand, inhibition of IRAK4 reduced, the production of TNF-α in macrophages after the incubation with *Lpb. plantarum* strains, although it was not statistically significant.

## Discussion

Understanding of the importance of fermented foods as a carrier of beneficial strains well-beyond their nutritional value has increased during the last century. This is in part due to recent studies in which the importance of diet in modulating gut microbiota and host physiology has been highlighted (Marco et al., 2017; Melini et al., 2019). In addition to the chemical composition of the food itself, the potential health advantages of fermented foods reside in resident microbes which may be consumed in large amounts. Recent studies have supported the potential benefits of these microbial strains due to their phylogenetic relationship with probiotic strains, predicting that their contribution to human health, could be similar to that of probiotics (Marco et al., 2017). However, there is still a lack of information regarding the mechanisms by which food resident microbes exert their functions and interact with host cells.

In accordance with the regulations outlined by FAO/WHO (2006), a potential probiotic strain should be able to survive in the gastrointestinal tract by exhibiting acid and bile tolerance, transiently colonize the gut and also be able to exert any benefits on the host (Hill et al., 2014). Moreover, probiotics should be harmless and not trigger any allergic response in the host (Sanders et al., 2010). Whilst such parameters are documented for probiotics used as functional food additives, the ability of food dwelling organisms to impact the host is less clear. In the current study we examined the interaction of *Lpb. plantarum* strains representative of natural strains consumed in different foods. Here we investigated the cellular location of *Lpb. plantarum* strains interacting with macrophages as well as their ability to stimulate cytokine/TLR expression and influence downstream signaling pathways.

The three *Lpb. plantarum* strains evaluated in this study have been tested for their ability to interact with human epithelial cells (Garcia-Gonzalez et al., 2018), to inhibit the biological activity of genotoxic compounds (Prete et al., 2017) and to survive bile-acid related stress (Prete et al., 2020). In view of their effects on the response of epithelial NCM460 cells (Garcia-Gonzalez et al., 2018), we evaluated their potential impact on immune system-cells using J774A.1 macrophages. Murine macrophages J774A.1 were selected as they express the PRRs involved in the recognition of bacterial pathogens and commensals and thereby mimic macrophages, mainly located in the lamina propria, which are adapted to remove pathogens and to maintain intestinal homeostasis (Ferreira dos Santos et al., 2016).

The cellular location of bacteria interacting with macrophages influences the triggering of specific PRRs (Figure 5A). We therefore evaluated the dynamics of bacterial interaction with macrophages in our assay system. Results showed that 4 h after incubation with bacteria, most of the total-associated bacteria corresponded to those phagocytosed by macrophages. Thus, the cross talk between macrophages and bacteria has the potential to be mediated by both extra- and intra-cellular PRRs (Philpott and Girardin, 2004; Fukata et al., 2009). In this study, we have focused on the two major groups of PRRs: TLRs and NLRs. TLRs are trans-membrane receptors, which can be either expressed on the cell surface or in intracellular organelles. TLRs differ from each other in ligand specificities, expression patterns, and in the target genes, and they can modulate (Janeway and Medzhitov, 2002) and play a critical role in the induction of immune responses (Macho Fernandez et al., 2011; Zhong et al., 2012). In particular TLR2, TLR4, TLR5, and TLR9 were selected here because of their involvement in recognition of bacteria associated molecular patterns (Konieczna et al., 2015; Wang et al., 2016). In contrast NOD1 and NOD2 (within the NLR family) act as cytoplasmic microbial sensors of intracellular bacteria recognizing peptidoglycan. The results show that *Lpb. plantarum* strains increase the expression of *Tlr2*, *Tlr9*, and *Nod2*. The profile of receptors activated was similar within the three *Lpb. plantarum* strains. The importance of TLR2 and NOD2 in recognizing bacteria has been previously described (Zeuthen et al., 2008; Jiang et al., 2013). The positive regulation of NOD2 by *Lpb. plantarum* in restoring homeostasis in germ-free mice highlights the interconnection between NOD2 and commensal bacteria (Petnicki-Ocwieja et al., 2009). Moreover, TLR2 seems to be crucial in the recognition of lactic acid bacteria, both in immune cells and epithelial cells where it has been implicated as a regulator of the epithelial integrity (Karczewski et al., 2010; Ren et al., 2016; Wang et al., 2019). While under inflammatory conditions it has been demonstrated that intestinal cells increase their expression of TLR2 and TLR4, the apical stimulation of TLR9 has been described to be anti-inflammatory (Lee et al., 2006).

In order to elucidate the immunomodulatory ability of *Lpb. plantarum* strains, the expression of a panel of inflammatory biomarkers was evaluated through qRT-PCR. Among the cytokines evaluated to measure the impact of *Lpb. plantarum* strains, TNF-α and IL-6 were selected as their release is directly induced by the activation of PRRs (Tanaka et al., 2014; Lee et al., 2016b). When PRRs are activated, they stimulate a range of signaling pathways, including NF-κB, and enhance the transcription of the mRNA of inflammatory cytokines such as TNF-α and IL-1ß. The release of TNF-α also activates the transcription factors to produce IL-6 (Tanaka et al., 2014). Cytokines, such as TNF-α and IL-6 play an important role in regulating immune response and inflammation (Luig et al., 2015; Lee et al., 2016b). Our results showed that there is an increase of TNF-α production induced by *Lpb. plantarum* strains but a weaker induction of IL-6. Indeed, Lactobacilli seem to be strong inducers of pro-inflammatory cytokines such as TNF-α (Matsubara et al., 2017; Štofilová et al., 2017). In particular, it has been proved that *Lpb. plantarum* LS/07 moderately induce pro-inflammatory cytokines from macrophages, whereas a higher induction of TNF-α release was observed after the co-culture of either monocyte/macrophages with the bacteria (Štofilová et al., 2017). However, like other probiotic properties described, the ability to modulate the immune system through the release of cytokines is strain-dependent, and thus results in literature can be contradictory (Lomax and Calder, 2009). The study conducted by Lee and colleagues underlined the different cytokine-inducing abilities of two *Lpb. plantarum* strains isolated from different sources. Differences in their capacity to stimulate cytokines and in the pattern of cytokines stimulated may be due to the structural differences in the cell wall of the bacteria. Small differences in the lipoteichoic acid and/or expression of different exopolysaccharides could lead to different biological functions (Lee et al., 2016a, b).

On the other hand, IL-6 is known to have both pro- and anti-inflammatory properties (Zielińska et al., 2019). The absence of IL-6 release by macrophages may help to convert an innate response into an adaptative response against pathogens (Gabay, 2006). This combined with the absence in TLR4 activation by *Lpb. plantarum* strains may reflect the anti-inflammatory effects of *Lpb. plantarum* strains. Since TLR4 signaling stimulates expression of genes encoding inflammatory molecules, a lack of activation of TLR4 by *Lpb. plantarum* strains favors an outcome that is less inflammatory, and may explain the reduced production of proinflammatory IL-6 as seen in our study.

We investigated whether cytokine production was linked to bacterial engagement with surface TLRs or intracellular NLRs in our system. Previous studies have shown that simultaneous activation of TLRs in macrophages leads to different cytokine expression profiles in comparison to stimulation of individual PRRs (Sato et al., 2000). TLRs share similar downstream signaling pathways *via* the NFκB cascade, through the adaptor proteins MyD88 firstly and subsequently IRAK4 (Chiu et al., 2013), whereas NOD1 and NOD2 use the RIP2 protein as an adaptor (Figure 5A). We used inhibitors of RIP2 and IRAK4 in our assay system and showed that a major decrease in TNF-α release was found following RIP2 inhibition. This implies that the majority of macrophage stimulation is via activation of the NLRs, a finding that reflects the fact that in our assay system, the majority of *Lpb. plantarum* strains are rapidly internalized by macrophages. Residual TNF-α release in the presence of the RIP2 inhibitor may be due to involvement of Myd88, illustrating that stimulation of macrophages by commensal bacteria is complex and multifactorial.

The immunomodulatory effects induced by food-related *Lpb. plantarum* strains may lead to enhancement of macrophage activation, however, further experiments will be necessary in order to establish the functional outcome resulting from conditioning of macrophages by *Lpb. plantarum* strains.

## Conclusion

Our findings showed that the two *Lpb. plantarum* strains which are representative of food-associated strains (Stanton et al., 1998; Perpetuini et al., 2020) were able to interact with macrophages to induce responses that are similar to a strain proposed to have probiotic potential. This paper indicates that internalized *Lpb. plantarum* strains are capable of stimulating macrophage responses through engagement of the NLR pathway, but are not cytotoxic under the conditions examined. As foods such as fermented table olives have been shown to contain high levels of *Lpb. plantarum* strains (represented by the C904 strain utilized here) the work suggests that autochthonous *Lpb. plantarum* from specific foods may have immune modulating effects when consumed in sufficient quantities.

## Data Availability Statement

The data available at: https://zenodo.org/record/3775511#.XqlxqZNKiS4.

## Author Contributions

AC, CG, and NB designed the study. NG-G, MAN-S, and MVR performed the experiments. NG-G, MAN-S, MVR, NB, CG, and AC analyzed the data, discussed the results, and drafted the manuscript. All the authors read and approved the final manuscript. All authors contributed to the article and approved the submitted version.

## Conflict of Interest

The authors declare that the research was conducted in the absence of any commercial or financial relationships that could be construed as a potential conflict of interest.

## Supplementary Material

Supplementary Figure 1 Light microscopy of *Lactiplantibacillus plantarum* cells and J774A.1 macrophages using Giemsa staining. **(A)** *Lpb. plantarum* LT52, IMC513, and C904 cells only. **(B)** *in vitro* exposure of J774A.1 to *Lpb. plantarum* LT52, IMC513, and C904 cells. Bacteria are phagocytosed and found inside macrophages cytoplasm, at least, 6 h post exposure.

## References

[B1] Bäuerl C. Llopis M. Antolín M. Monedero V. Mata M. Zúñiga M. (2013). *Lactobacillus paracasei* and *Lactobacillus plantarum* strains downregulate proinflammatory genes in an *ex vivo* system of cultured human colonic mucosa. *Genes Nutr.* 8 165–180. 10.1007/s12263-012-0301-y 22669626 PMC3575885

[B2] Behera S. S. Ray R. C. Zdolec N. (2018). *Lactobacillus plantarum* with functional properties: an approach to increase safety and shelf-life of fermented foods. *Biomed. Res. Int.* 2018:9361614. 10.1155/2018/9361614 29998137 PMC5994577

[B3] Brown R. L. Sequeira R. P. Clarke T. B. (2017). The microbiota protects against respiratory infection via GM-CSF signaling. *Nat. Commun.* 8:1512. 10.1038/s41467-017-01803-x 29142211 PMC5688119

[B4] Cani P. D. Bibiloni R. Knauf C. Waget A. Neyrinck A. M. Delzenne N. M. (2008). Changes in gut microbiota control metabolic endotoxemia-induced inflammation in high-fat diet-induced obesity and diabetes in mice. *Diabetes* 57 1470–1481. 10.2337/db07-1403 18305141

[B5] Chiu Y. H. Lu Y. C. Ou C. C. Lin S. L. Tsai C. C. Huang C. T. (2013). *Lactobacillus plantarum* MYL26 induces endotoxin tolerance phenotype in Caco-2 cells. *BMC Microbiol.* 13:190. 10.1186/1471-2180-13-190 23937116 PMC3751156

[B6] Corsetti A. Prete R. Garcia-Gonzalez N. (2018). “Lactic acid bacteria: *Lactobacillus* spp. *Lactobacillus plantarum*,” in *Reference Module in Food Science*, ed. Elsevier (New York, NY: Elsevier), 1–8.

[B7] Corthésy B. Gaskins H. R. Mercenier A. (2007). Cross-talk between probiotic bacteria and the host immune system. *J. Nutr.* 137(3 Suppl. 2) 781S–790S. 10.1093/jn/137.3.781S 17311975

[B8] de Vrese M. Schrezenmeir J. (2008). Probiotics, prebiotics, and synbiotics. *Adv. Biochem. Eng. Biotechnol.* 111 1–66. 10.1007/10_2008_097 18461293

[B9] Dong H. Rowland I. Tuohy K. M. Thomas L. V. Yaqoob P. (2010). Selective effects of *Lactobacillus casei* Shirota on T cell activation, natural killer cell activity and cytokine production. *Clin. Exp. Immunol.* 161 378–388. 10.1111/j.1365-2249.2010.04173.x 20456417 PMC2909421

[B10] FAO/WHO (2006). *Probiotics in Food. Health and Nutritional Properties and Guidelines for Evaluation.* Rome: FAO Food Nutrition.

[B11] Ferreira dos Santos T. Alves Melo T. Evangelista Almeida M. Passos Rezende R. Cristina Romano C. (2016). Immunomodulatory effects of *Lactobacillus plantarum* Lp62 on intestinal epithelial and mononuclear cells. *Biomed. Res. Int.* 2016:8404156. 10.1155/2016/8404156 27446958 PMC4944036

[B12] Fukata M. Vamadevan A. S. Abreu M. T. (2009). Toll-like receptors (TLRs) and Nod-like receptors (NLRs) in inflammatory disorders. *Semin. Immunol.* 21 242–253. 10.1016/j.smim.2009.06.005 19748439

[B13] Gabay C. (2006). Interleukin-6 and chronic inflammation. *Arthritis Res. Ther.* 8(Suppl. 2):S3. 10.1186/ar1917 16899107 PMC3226076

[B14] Garcia-Gonzalez N. Prete R. Battista N. Corsetti A. (2018). Adhesion properties of food-associated *Lactobacillus plantarum* strains on human intestinal cells and modulation of IL-8 release. *Front. Microbiol.* 9:2392. 10.3389/fmicb.2018.02392 30349520 PMC6186789

[B15] Ghadimi D. Vrese M. Heller K. J. Schrezenmeir J. (2010). Effect of natural commensal-origin DNA on toll-like receptor 9 (TLR9) signaling cascade, chemokine IL-8 expression, and barrier integritiy of polarized intestinal epithelial cells. *Inflamm. Bowel Dis.* 16 410–427. 10.1002/ibd.21057 19714766

[B16] Heperkan D. (2013). Microbiota of table olive fermentations and criteria of selection for their use as starters. *Front. Microbiol.* 4:143. 10.3389/fmicb.2013.00143 23781216 PMC3679444

[B17] Hill C. Guarner F. Reid G. Gibson G. R. Merenstein D. J. Pot B. (2014). Expert consensus document. The international scientific association for probiotics and prebiotics consensus statement on the scope and appropriate use of the term probiotic. *Nat. Rev. Gastroenterol. Hepatol.* 11 506–514. 10.1038/nrgastro.2014.66 24912386

[B18] Hurtado A. Reguant C. Bordons A. Rozès N. (2012). Lactic acid bacteria from fermented table olives. *Food Microbiol.* 31 1–8. 10.1016/j.fm.2012.01.006 22475936

[B19] Ivec M. Botić T. Koren S. Jakobsen M. Weingartl H. Cencic A. (2007). Interactions of macrophages with probiotic bacteria lead to increased antiviral response against vesicular stomatitis virus. *Antiviral Res.* 75 266–274. 10.1016/j.antiviral.2007.03.013 17512614

[B20] Janeway C. A. Medzhitov R. (2002). Innate immune recognition. *Annu. Rev. Immunol.* 20 197–216. 10.1146/annurev.immunol.20.083001.084359 11861602

[B21] Jiang W. Wang X. Zeng B. Liu L. Tardivel A. Wei H. (2013). Recognition of gut microbiota by NOD2 is essential for the homeostasis of intestinal intraepithelial lymphocytes. *J. Exp. Med.* 210 2465–2476. 10.1084/jem.20122490 24062413 PMC3804938

[B22] Kang H. J. Im S. H. (2015). Probiotics as an immune modulator. *J. Nutr. Sci. Vitaminol. (Tokyo)* 61 (Suppl.) S103–S105. 10.3177/jnsv.61.S103 26598815

[B23] Karczewski J. Troost F. J. Konings I. Dekker J. Kleerebezem M. Brummer R. J. (2010). Regulation of human epithelial tight junction proteins by *Lactobacillus plantarum in vivo* and protective effects on the epithelial barrier. *Am. J. Physiol. Gastrointest. Liver Physiol.* 298 G851–G859. 10.1152/ajpgi.00327.2009 20224007

[B24] Konieczna P. Schiavi E. Ziegler M. Groeger D. Healy S. Grant R. (2015). Human dendritic cell DC-SIGN and TLR-2 mediate complementary immune regulatory activities in response to *Lactobacillus rhamnosus* JB-1. *PLoS One* 10:e0120261. 10.1371/journal.pone.0120261 25816321 PMC4376398

[B25] Lee I. C. Caggianiello G. van Swam I. I. Taverne N. Meijerink M. Bron P. A. (2016a). Strain-specific features of extracellular polysaccharides and their impact on *Lactobacillus plantarum*-host interactions. *Appl. Environ. Microbiol.* 82 3959–3970. 10.1128/AEM.00306-16 27107126 PMC4907176

[B26] Lee J. Mo J. H. Katakura K. Alkalay I. Rucker A. N. Liu Y. T. (2006). Maintenance of colonic homeostasis by distinctive apical TLR9 signalling in intestinal epithelial cells. *Nat. Cell Biol.* 8 1327–1336. 10.1038/ncb1500 17128265

[B27] Lee Y. D. Hong Y. F. Jeon B. Jung B. J. Chung D. K. Kim H. (2016b). Differential cytokine regulatory effect of three *Lactobacillus* strains isolated from fermented foods. *J. Microbiol. Biotechnol.* 26 1517–1526. 10.4014/jmb.1601.01044 27221109

[B28] Lomax A. R. Calder P. C. (2009). Probiotics, immune function, infection and inflammation: a review of the evidence from studies conducted in humans. *Curr. Pharm. Des.* 15 1428–1518. 10.2174/138161209788168155 19442167

[B29] Luig M. Kluger M. A. Goerke B. Meyer M. Nosko A. Yan I. (2015). Inflammation-induced IL-6 functions as a natural brake on macrophages and limits GN. *J. Am. Soc. Nephrol.* 26 1597–1607. 10.1681/ASN.2014060620 25655068 PMC4483592

[B30] Macho Fernandez E. Valenti V. Rockel C. Hermann C. Pot B. Boneca I. G. (2011). Anti-inflammatory capacity of selected lactobacilli in experimental colitis is driven by NOD2-mediated recognition of a specific peptidoglycan-derived muropeptide. *Gut* 60 1050–1059. 10.1136/gut.2010.232918 21471573

[B31] Marco M. L. Heeney D. Binda S. Cifelli C. J. Cotter P. D. Foligné B. (2017). Health benefits of fermented foods: microbiota and beyond. *Curr. Opin. Biotechnol.* 44 94–102. 10.1016/j.copbio.2016.11.010 27998788

[B32] Markowiak P. Śliżewska K. (2017). Effects of probiotics, prebiotics, and synbiotics on human health. *Nutrients* 9:E1021. 10.3390/nu9091021 28914794 PMC5622781

[B33] Matsubara V. H. Ishikawa K. H. Ando-Suguimoto E. S. Bueno-Silva B. Nakamae A. E. M. Mayer M. P. A. (2017). Probiotic bacteria alter pattern-recognition receptor expression and cytokine profile in a human macrophage model challenged with *Candida albicans* and lipopolysaccharide. *Front. Microbiol.* 8:2280. 10.3389/fmicb.2017.02280 29238325 PMC5712552

[B34] Melini F. Melini V. Luziatelli F. Ficca A. G. Ruzzi M. (2019). Health-promoting components in fermented foods: an up-to-date systematic review. *Nutrients* 11:1189. 10.3390/nu11051189 31137859 PMC6567126

[B35] Melmed G. Thomas L. S. Lee N. Tesfay S. Y. Lukasek K. Michelsen K. S. (2003). Human intestinal epithelial cells are broadly unresponsive to Toll-like receptor 2-dependent bacterial ligands: implications for host-microbial interactions in the gut. *J. Immunol.* 170 1406–1415. 10.4049/jimmunol.170.3.1406 12538701

[B36] Paolillo R. Romano Carratelli C. Sorrentino S. Mazzola N. Rizzo A. (2009). Immunomodulatory effects of *Lactobacillus plantarum* on human colon cancer cells. *Int. Immunopharmacol.* 9 1265–1271. 10.1016/j.intimp.2009.07.008 19647100

[B37] Pathmakanthan S. Li C. K. Cowie J. Hawkey C. J. (2004). *Lactobacillus plantarum* 299: beneficial in vitro immunomodulation in cells extracted from inflamed human colon. *J. Gastroenterol. Hepatol.* 19 166–173. 10.1111/j.1440-1746.2004.03181.x 14731126

[B38] Perpetuini G. Prete R. Garcia-Gonzalez N. Khairul Alam M. Corsetti A. (2020). Table olives more than a fermented food. *Foods* 9:E178. 10.3390/foods9020178 32059387 PMC7073621

[B39] Petnicki-Ocwieja T. Hrncir T. Liu Y. J. Biswas A. Hudcovic T. Tlaskalova-Hogenova H. (2009). Nod2 is required for the regulation of commensal microbiota in the intestine. *Proc. Natl. Acad. Sci. U.S.A.* 106 15813–15818. 10.1073/pnas.0907722106 19805227 PMC2747201

[B40] Philpott D. J. Girardin S. E. (2004). The role of Toll-like receptors and Nod proteins in bacterial infection. *Mol. Immunol.* 41 1099–1108. 10.1016/j.molimm.2004.06.012 15476921

[B41] Plaza-Diaz J. Ruiz-Ojeda F. J. Gil-Campos M. Gil A. (2019). Mechanisms of action of probiotics. *Adv. Nutr.* 10 S49–S66. 10.1093/advances/nmy063 30721959 PMC6363529

[B42] Plot B. Felis G. De Bruyne K. Tsakalidou E. Papadimitriou K. Leisner J. (2014). *Lactic Acid Bacteria: Biodiversity and Taxonomy*, eds Holzapfel W. H. Wood B. J. B. (Hoboken, NJ: John Wiley & Sons).

[B43] Prete R. Long S. L. Gallardo A. L. Gahan C. G. Corsetti A. Joyce S. A. (2020). Beneficial bile acid metabolism from *Lactobacillus plantarum* of food origin. *Sci. Rep.* 10:1165. 10.1038/s41598-020-58069-5 31980710 PMC6981223

[B44] Prete R. Tofalo R. Federici E. Ciarrocchi A. Cenci G. Corsetti A. (2017). Food-associated. *Front. Microbiol.* 8:2349. 10.3389/fmicb.2017.02349 29234315 PMC5712336

[B45] Ren C. Zhang Q. de Haan B. J. Zhang H. Faas M. M. de Vos P. (2016). Identification of TLR2/TLR6 signalling lactic acid bacteria for supporting immune regulation. *Sci. Rep.* 6:34561. 10.1038/srep34561 27708357 PMC5052581

[B46] Ryan P. M. Stolte E. H. London L. E. E. Wells J. M. Long S. L. Joyce S. A. (2019). *Lactobacillus mucosae* DPC 6426 as a bile-modifying and immunomodulatory microbe. *BMC Microbiol.* 19:33. 10.1186/s12866-019-1403-0 30736731 PMC6368806

[B47] Sanders M. E. Akkermans L. M. Haller D. Hammerman C. Heimbach J. Hörmannsperger G. (2010). Safety assessment of probiotics for human use. *Gut Microbes* 1 164–185. 10.4161/gmic.1.3.12127 21327023 PMC3023597

[B48] Sato S. Nomura F. Kawai T. Takeuchi O. Mühlradt P. F. Takeda K. (2000). Synergy and cross-tolerance between toll-like receptor (TLR) 2- and TLR4-mediated signaling pathways. *J. Immunol.* 165 7096–7101. 10.4049/jimmunol.165.12.7096 11120839

[B49] Seddik H. A. Bendali F. Gancel F. Fliss I. Spano G. Drider D. (2017). *Lactobacillus plantarum* and its probiotic and food potentialities. *Probiotics Antimicrob. Proteins* 9 111–122. 10.1007/s12602-017-9264-z 28271469

[B50] Seganish W. M. (2016). Inhibitors of interleukin-1 receptor-associated kinase 4 (IRAK4): a patent review (2012-2015). *Expert Opin. Ther. Pat.* 26 917–932. 10.1080/13543776.2016.1202926 27310003

[B51] Stanton C. Gardiner G. Lynch P. Collins J. Fitzgerald G. Ross R. (1998). Probiotic cheese. *Int. Dairy J.* 8 491–496.

[B52] Štofilová J. Langerholc T. Botta C. Treven P. Gradišnik L. Salaj R. (2017). Cytokine production *in vitro* and in rat model of colitis in response to *Lactobacillus plantarum* LS/07. *Biomed. Pharmacother.* 94 1176–1185. 10.1016/j.biopha.2017.07.138 28830068

[B53] Suzuki E. Umezawa K. (2006). Inhibition of macrophage activation and phagocytosis by a novel NF-kappaB inhibitor, dehydroxymethylepoxyquinomicin. *Biomed. Pharmacother.* 60 578–586. 10.1016/j.biopha.2006.07.089 16978829

[B54] Tallon R. Arias S. Bressollier P. Urdaci M. C. (2007). Strain- and matrix-dependent adhesion of *Lactobacillus plantarum* is mediated by proteinaceous bacterial compounds. *J. Appl. Microbiol.* 102 442–451. 10.1111/j.1365-2672.2006.03086.x 17241350

[B55] Tanaka T. Narazaki M. Kishimoto T. (2014). IL-6 in inflammation, immunity, and disease. *Cold Spring Harb. Perspect. Biol.* 6:a016295. 10.1101/cshperspect.a016295 25190079 PMC4176007

[B56] Vargas García C. E. Petrova M. Claes I. J. De Boeck I. Verhoeven T. L. Dilissen E. (2015). Piliation of *Lactobacillus rhamnosus* GG promotes adhesion, phagocytosis, and cytokine modulation in macrophages. *Appl. Environ. Microbiol.* 81 2050–2062. 10.1128/AEM.03949-14 25576613 PMC4345371

[B57] Wang J. Zhang W. Wang S. Liu H. Zhang D. Wang Y. (2019). Swine-derived probiotic *Lactobacillus plantarum* modulates porcine intestinal endogenous host defense peptide synthesis through TLR2/MAPK/AP-1 signaling pathway. *Front. Immunol.* 10:2691. 10.3389/fimmu.2019.02691 31803195 PMC6877743

[B58] Wang Y. Xie J. Li Y. Dong S. Liu H. Chen J. (2016). Probiotic *Lactobacillus casei* Zhang reduces pro-inflammatory cytokine production and hepatic inflammation in a rat model of acute liver failure. *Eur. J. Nutr.* 55 821–831. 10.1007/s00394-015-0904-3 25893720

[B59] Weibel G. L. Joshi M. R. Jerome W. G. Bates S. R. Yu K. J. Phillips M. C. (2012). Cytoskeleton disruption in J774 macrophages: consequences for lipid droplet formation and cholesterol flux. *Biochim. Biophys. Acta* 1821 464–472. 10.1016/j.bbalip.2011.09.015 22015387 PMC3274585

[B60] Wells J. M. (2011). Immunomodulatory mechanisms of lactobacilli. *Microb. Cell Fact.* 10:S17. 10.1186/1475-2859-10-S1-S17 21995674 PMC3231924

[B61] Zeuthen L. H. Fink L. N. Frøkiaer H. (2008). Toll-like receptor 2 and nucleotide-binding oligomerization domain-2 play divergent roles in the recognition of gut-derived lactobacilli and bifidobacteria in dendritic cells. *Immunology* 124 489–502. 10.1111/j.1365-2567.2007.02800.x 18217947 PMC2492941

[B62] Zheng J. Wittouck S. Salvetti E. Franz C. M. A. P. Harris H. M. B. Mattarelli P. (2020). A taxonomic note on the genus *Lactobacillus*: description of 23 novel genera, emended description of the genus *Lactobacillus Beijerinck* 1901, and union of *Lactobacillaceae* and *Leuconostocaceae*. *Int. J. Syst. Evol. Microbiol.* 70 2782–2858. 10.1099/ijsem.0.004107 32293557

[B63] Zhong Y. Huang J. Tang W. Chen B. Cai W. (2012). Effects of probiotics, probiotic DNA and the CpG oligodeoxynucleotides on ovalbumin-sensitized Brown-Norway rats via TLR9/NF-κB pathway. *FEMS Immunol. Med. Microbiol.* 66 71–82. 10.1111/j.1574-695X.2012.00991.x 22612777

[B64] Zielińska D. Długosz E. Zawistowska-Deniziak A. (2019). Functional properties of food origin *Lactobacillus* in the gastrointestinal ecosystem-*In Vitro* study. *Probiotics Antimicrob. Proteins* 11 820–829. 10.1007/s12602-018-9458-z 30141062 PMC6695375

