# Supplementary figures and images for: Immunomodulation of J774A.1 Murine Macrophages by Lactiplantibacillus plantarum Strains Isolated From the Human Gastrointestinal Tract and Fermented Foods
Source: Front Microbiol. 2021 Jan 12;11:557143. doi: 10.3389/fmicb.2020.557143 (PMC7835322; doi:10.3389/fmicb.2020.557143)

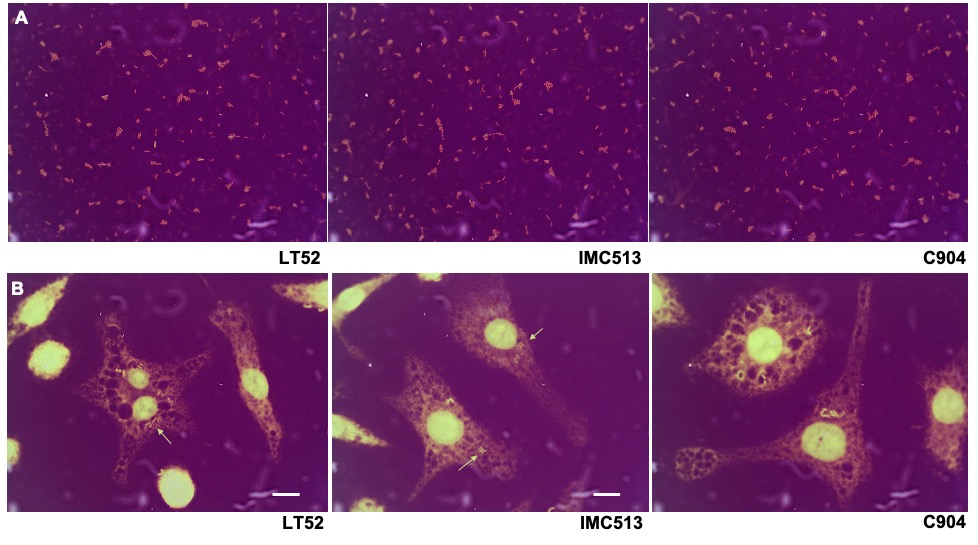

Supplement: Supplementary Figure 1 — Light microscopy of Lactiplantibacillus plantarum cells and J774A.1 macrophages using Giemsa staining. (A) Lpb. plantarum LT52, IMC513, and C904 cells only. (B) in vitro exposure of J774A.1 to Lpb. plantarum LT52, IMC513, and C904 cells. Bacteria are phagocytosed and found inside macrophages cytoplasm, at least, 6 h post exposure. [file Image_1.JPEG]
